# Evidence for negative selection of gene variants that increase dependence on dietary choline in a Gambian cohort

**DOI:** 10.1096/fj.15-271056

**Published:** 2015-04-28

**Authors:** Matt J. Silver, Karen D. Corbin, Garrett Hellenthal, Kerry-Ann da Costa, Paula Dominguez-Salas, Sophie E. Moore, Jennifer Owen, Andrew M. Prentice, Branwen J. Hennig, Steven H. Zeisel

**Affiliations:** *Medical Research Council International Nutrition Group, London School of Hygiene and Tropical Medicine, London, United Kingdom; ^†^Medical Research Council Unit, Banjul, The Gambia; ^‡^Nutrition Research Institute, North Carolina Research Campus, Kannapolis, North Carolina, USA; ^§^Department of Nutrition, Gillings School of Global Public Health, University of North Carolina at Chapel Hill, Chapel Hill, North Carolina, USA; ^¶^University College London Genetics Institute, University College London, United Kingdom; ^‖^Toxicology Services, Incorporated, Chapel Hill, North Carolina, USA; and ^#^Maternal and Child Nutrition Group, Medical Research Council Human Nutrition Research, Cambridge, United Kingdom

**Keywords:** diet and selection, adequate intake levels, phosphatidylethanolamine-*N*-methyltransferase, choline dehydrogenase, methylenetetrahydrofolate dehydrogenase

## Abstract

Choline is an essential nutrient, and the amount needed in the diet is modulated by several factors. Given geographical differences in dietary choline intake and disparate frequencies of single-nucleotide polymorphisms (SNPs) in choline metabolism genes between ethnic groups, we tested the hypothesis that 3 SNPs that increase dependence on dietary choline would be under negative selection pressure in settings where choline intake is low: choline dehydrogenase (*CHDH*) rs12676, methylenetetrahydrofolate reductase 1 (*MTHFD1*) rs2236225, and phosphatidylethanolamine-*N*-methyltransferase (*PEMT*) rs12325817. Evidence of negative selection was assessed in 2 populations: one in The Gambia, West Africa, where there is historic evidence of a choline-poor diet, and the other in the United States, with a comparatively choline-rich diet. We used 2 independent methods, and confirmation of our hypothesis was sought *via* a comparison with SNP data from the Maasai, an East African population with a genetic background similar to that of Gambians but with a traditional diet that is higher in choline. Our results show that frequencies of SNPs known to increase dependence on dietary choline are significantly reduced in the low-choline setting of The Gambia. Our findings suggest that adequate intake levels of choline may have to be reevaluated in different ethnic groups and highlight a possible approach for identifying novel functional SNPs under the influence of dietary selective pressure.—Silver, M. J., Corbin, K. D., Hellenthal, G., da Costa, K.-A., Dominguez-Salas, P., Moore, S. E., Owen, J., Prentice, A. M., Hennig, B. J., Zeisel, S. H. Evidence for negative selection of gene variants that increase dependence on dietary choline in a Gambian cohort.

Choline is an essential nutrient (1) with functional relevance in a wide array of biologic pathways, including epigenetic modulation of gene expression, brain development, hepatic lipid homeostasis, and energy metabolism. Choline is positioned at the intersection of 1-carbon metabolism pathways, which generate methyl groups from choline, methionine, and folate that are essential for biologic methylation reactions (2). Two key phenotypes emerge when dietary choline is limited in humans. The most prominent is in the liver, where accumulation of lipids is concurrent with increased markers of damage, such as elevated serum liver enzymes and hepatocyte apoptosis. A smaller subset of individuals exhibit a muscle phenotype characterized by elevated serum creatine phosphokinase from muscle. These symptoms resolve when choline is reintroduced into the diet (3–6). Furthermore, there is an extensive body of literature demonstrating the metabolic and health consequences of inadequate choline intake, ranging from neural tube defects to cancer, in various ethnic groups (3, 6–11).

Adequate intake (AI) for choline, established from observations of choline intake in healthy U.S. adults, is 425–550 mg/d (1, 12). However, the requirement for choline is modulated by several factors, including sex, menopausal status (5), and the gut microbiome (13). Genetic variation also plays a role, and 3 functional single-nucleotide polymorphisms (SNPs) in particular are known to increase dependence on dietary choline. These are hereafter referred to as choline-dependent (CD) SNPs: choline dehydrogenase (*CHDH*), rs12676; methylenetetrahydrofolate reductase 1 (*MTHFD1*), rs2236225; and phosphatidylethanolamine-*N*-methyltransferase (*PEMT*), rs12325817 (3, 6) (**Fig. 1**).

**Figure 1.**
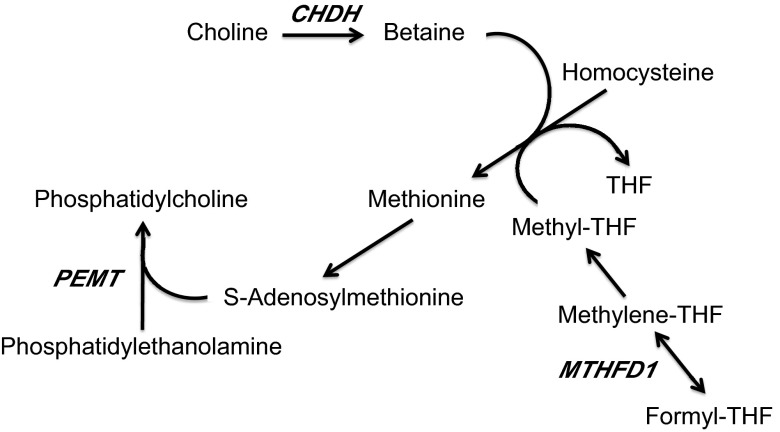
Metabolic pathways modulated by *CHDH*, *PEMT*, and *MTHFD1*. Choline is oxidized to form betaine by CHDH. Betaine is used as a methyl donor in the formation of methionine. MTHFD catalyzes the formation of methyltetrahydrofolate, which is an alternative methyl donor in the formation of methionine. Methionine is used to form *S*-adenosylmethionine, which is necessary in the methylation of phosphatidylethanolamine to form phosphatidylcholine. Genetic polymorphisms in *CHDH*, *PEMT*, and *MTHFD1* increase dependence on dietary choline by modulating the formation of choline and its utilization as a methyl donor.

Several lines of evidence demonstrate a role for CD SNPs in affecting metabolism and dependence on dietary choline. *CHDH* encodes a mitochondrial protein that catalyzes the first irreversible step in the oxidation of choline to betaine. Premenopausal female carriers of the T allele of *CHDH* rs12676 (a nonsynonymous coding SNP) have greater dependence on dietary choline (3). In men, this allele is also associated with lower sperm CHDH protein levels (14). Individuals with this SNP need more choline precursor to drive production of this reaction’s product, betaine, which is necessary for methylation reactions.

*MTHFD1* encodes a folate-metabolizing enzyme that catalyzes 3 reactions that direct the flow of 1-carbon folates (15); the formation of 5-methyl-terahydrofolate (5-methyl-THF) is practically irreversible *in vivo*, but the interconversion of 5,10-methylene-THF and 10-formyl-THF is closer to equilibrium (6, 16). Thus, 5,10-methylene-THF may be directed by MTHFD1, either toward homocysteine methylation or away from it. The *MTHFD1* rs2236225 polymorphism (a nonsynonymous coding SNP) increases the flux between 5,10-methylene-THF and 10-formyl-THF and thereby reduces the flux between 5,10-methylene-THF and 5-methyl-THF, making less 5-methyl-THF available for homocysteine remethylation. When 5-methyl-THF is not available, more betaine from choline is needed for homocysteine remethylation (6, 17). Carriers of the A allele of *MTHFD1* rs2236225 thus have an increased dependence on dietary choline (6).

*PEMT* encodes an enzyme that sequentially methylates phosphatidylethanolamine to generate phosphatidylcholine, a source of choline (18). *PEMT* expression is induced by estrogen, and *PEMT* rs12325817 is a promoter SNP that abrogates estrogen-mediated induction of the gene (19). Female carriers of the C allele of this SNP (on the coding strand) are more susceptible to development of organ dysfunction when eating a low-choline diet (3–5), because they are less able to induce the gene with estrogen and thereby make less of their own choline (in the form of phosphatidylcholine). It is reasonable to suggest that women with CD SNPs who are eating low-choline diets deliver less choline to the fetus (*via* the placenta) and that this could negatively affect fetal outcome (20). There may also be effects on the establishment of methylation patterns in the epigenome of the very early embryo, in that these are known to be sensitive to nutrients in the 1-carbon pathway (21).

The distribution of multiple SNPs in genes within the 1-carbon metabolism pathway varies across different ethnic groups, and these genetic patterns are associated with different health outcomes (22, 23). Differences in the distribution of CD SNPs are particularly evident between populations of Caucasian and African descent (22, 23). The diversity in access to choline in various regions of the world led us to hypothesize that the disparate frequency of functional variants in choline metabolism is influenced by dietary selective pressures. Using 2 independent statistical methods, we tested this hypothesis of choline-mediated selective pressure by comparing 2 populations: one in The Gambia (GAM) with a choline-poor diet (24–28), and the other composed of individuals of Caucasian/European descent (EUR) from North Carolina in the United States, with a relatively choline-rich diet (29–32). Furthermore, we compared allele frequencies of CD SNPs in GAM and EUR cohorts with those observed in another African population [HapMap (International HapMap Project, National Center for Biotechnology Information, Bethesda, MD, USA)]: the Maasai in Kinyawa, Kenya; MKK), an ethnic population that is genetically more similar to Gambians, but with a traditional diet that is relatively high in choline (33).

## MATERIALS AND METHODS

### North Carolina clinical cohort

The individuals included in this study were men and women from 3 previously reported studies (4, 5, 34). Briefly, these studies examined the amount of dietary choline needed for optimal health and the role played by genetic variation. In one study, dietary choline restriction produced liver and muscle phenotypes in subjects who were inpatients at the Clinical and Translational Research Center, University of North Carolina (UNC) Chapel Hill School of Medicine. There were 3 phases to the study. The baseline phase provided a diet with adequate choline (550 mg/70 kg per day). The choline depletion phase provided 50 mg choline per day. The final repletion phase reintroduced adequate choline into the diets (5). The second study was similar to the first, but the focus was on women and the importance of estrogen for endogenous choline synthesis (4). In the third study, pregnant women were examined to determine whether total choline intake, SNPs, or both influence the amount of choline and its metabolites found in breast milk and plasma (34). Written informed consent was obtained from all participants, and the Institutional Review Board at UNC Chapel Hill approved all protocols. The samples used in the study included 162 Caucasian individuals from whom sufficient DNA was available for genotyping. Three first-degree relatives were excluded, leaving 159 subjects (17 males and 142 females) for analysis.

### Gambian study cohort

We selected women who participated in 1 of 3 studies in The Gambia (24, 35, 36), for whom a DNA sample was available for genotyping and excluded all first-degree relatives, so that 241 subjects were available for the study. Briefly, all women were recruited between 2009 and 2010 in the Kiang West district of rural Gambia, from the 36 villages in the catchment area of the Medical Research Center (MRC) International Nutrition Group’s field station at MRC Keneba (*http://www.ing.mrc.ac.uk*). Written informed consent was obtained from all participants, and the joint Gambian Government/MRC Ethics Committee approved all procedures.

### Gene and variant selection and genotyping

Gene variants used in this study were those selected for a previous investigation that targeted SNP mapping to genes in the choline pathway and the intersecting folate and methionine pathways (±5 kb from gene boundaries, to assess the role of distal regulatory elements); in peripherally related genes that metabolize choline containing lipids; or in genes with a direct relationship to fatty liver, a choline-mediated phenotype (22). The set of genotyped SNPs included the 3 CD SNPs that were the focus of this study (*CHDH* rs12676, *MTHFD1* rs2236225, and *PEMT* rs12325817), because they have been associated with an increased dependence on dietary choline (3–6, 34, 37) and have known functional effects on choline metabolism (14, 15, 19, 37, 38). For this study, we included 226 SNPs genotyped in both the GAM and EUR cohorts, but removed 12 SNPs for which there is limited evidence of an influence on dietary choline requirements (23), but no functional data, as these may otherwise have biased our analysis. Thus, of the remaining 214 SNPs, 3 are the CD SNPs and the remainder lack any published evidence of a role in modulating choline requirements, as is necessary for our statistical tests to be valid. Details of further SNP filtering procedures are given below.

Samples were genotyped as described in several publications (6, 19, 22, 23). Briefly, 98% of SNPs were genotyped with an oligo-specific extension-ligation assay on a custom Golden Gate array (Illumina, Inc., San Diego, CA, USA) (39). We used an in-house real-time PCR assay for the *PEMT* rs12325817 SNP (22, 23), because it cannot be genotyped on the Illumina platform. Four SNPs in the EUR cohort were genotyped by alternative methods. Two CD SNPs, rs12676 and rs2236225, had a subset of samples that failed on the Illumina platform, so they were genotyped *via* matrix-assisted laser desorption/ionization–time-of-flight (MALDI-TOF) primer-extension assay (Sequenom, Inc., San Diego, CA, USA) (22). Two other SNPs, rs3733890 and rs4244599, were part of targeted investigations before implementation of the custom Illumina array. They were genotyped *via* MALDI-TOF mass spectrometry and real-time PCR, respectively, as described elsewhere (6, 19).

### MKK genotypes

We downloaded MKK genotypes for 95 unrelated individuals (42 males, 43 females), genotyped at 1,457,897 SNPs as part of HapMap3 (40). A majority of the 214 study SNPs genotyped in GAM and EUR were not present in the HapMap data, including 2 of the 3 CD SNPs. All missing SNPs were therefore imputed using IMPUTE2 (41), with phase 1 data from the 1000 Genomes project (EMBL-EBI, Hinxton, United Kingdom) as a reference panel. HapMap MKK genotypes were converted from the hg18 to hg19 genome build using liftOver (*http://hgdownload.cse.ucsc.edu/admin/exe/linux.x86_64/liftover*) before imputation. Metrics for imputation quality indicated that the 2 CD SNPs were imputed with high confidence (IMPUTE2 info = 0.98 and certainty = 0.99 for rs12676; info = 0.97, certainty = 0.99 for rs12325817). IMPUTE2 metrics for internal cross-validation of existing sample genotypes against imputed values indicated that imputation was successful (>95% overall concordance; Supplemental Table 1). Thirty-four SNPs could not be confidently aligned with GAM and EUR allele calls because they had complementary alleles that made strand direction difficult to assign. Thus, 180 SNPs remained for the MKK cohort before SNP filtering.

### SNP filtering

Our statistical tests for selection treat missing and monomorphic SNPs differently and perform different cross-cohort comparisons. For this reason, SNP filtering strategies vary, and we consider these for each test separately.

#### Method 1: pairwise cross-cohort comparisons

For each cross-cohort comparison, only SNPs with genotype data across both cohorts were considered (GAM *vs.* EUR: 214 SNPs considered; GAM *vs.* MKK and MKK *vs.* EUR: 180). All SNPs with a genotype call rate <90% in either cohort were removed (GAM *vs.* EUR: 3 SNPs removed; GAM *vs.* MKK 2; MKK *vs.* EUR: 1). Because nonzero minor allele frequencies (MAFs) are necessary to calculate variance-adjusted statistics, we further removed all SNPs that were monomorphic in either cohort (GAM *vs.* EUR: 16 SNPs removed; GAM *vs.* MKK: 6 SNPs; MKK *vs.* EUR: 12 SNPs). Finally, because the statistical test assumes that SNPs are independent, for each cross-cohort comparison, we measured pairwise correlations between all SNPs in each cohort and retained only 1 of each pair of SNPs with an *r*^2^ ≥ 0.8 in either cohort (GAM *vs*. EUR: 21 SNPs removed; GAM *vs.* MKK 23 SNPs; MKK *vs.* EUR: 26 SNPs). This process left 174 SNPs for the GAM *vs.* EUR analysis, 149 SNPs for GAM *vs.* MKK and 141 SNPs for MKK *vs.* EUR.

#### Method 2: population genetic model

This method can accommodate SNPs that are missing in only 1 cohort or are monomorphic in 1–2 of the 3 cohorts. We therefore considered all 214 SNPs for this analysis, but recorded SNPs with a genotype call rate <90% in any cohort as missing for that cohort (1 EUR SNP and 2 GAM SNPs). We further removed 2 SNPs that were monomorphic across all 3 cohorts and performed linkage disequilibrium (LD) filtering across all 3 cohorts using the same *r*^2^ threshold as described for method 1, which resulted in the removal of another 38 SNPs, leaving 174 SNPs for the method 2 analysis. To generate empirical probabilities to test against the null hypothesis of no negative selection in the GAM cohort at the CD SNPs, we used 144 of these 174 SNPs that were nonmissing in all 3 cohorts. However, we note that results were very similar when we used all 210 SNPs that were nonmissing in the Gambia cohort for this analysis.

### Statistical tests for selection

Variation in SNP allele frequencies, both within and between populations, may be driven by selection or by random processes of genetic drift. Genetic drift can lead to SNPs being driven to fixation or lost entirely from a population simply by random chance (42). It is also possible for variants to arise *de novo* in a population through mutation. It is therefore important to allow for the possibility that any or all of these factors may be the cause of variation in allele frequencies when looking for evidence of selection at any particular SNP. We used 2 statistical tests for assessing evidence of negative selection at CD SNPs in the GAM sample.

#### Method 1: pairwise cross-cohort comparisons

Methods for assessing evidence of selection generally rely on dense genotyping around SNPs or genes of interest (43). Because we did not have access to such data, we instead tested each SNP independently, using a statistical test that compares allele frequency changes of CD SNPs to an empirical null distribution of the same test statistic calculated for other genotyped SNPs not known to increase dependence on dietary choline. We performed 3 separate cross-cohort comparisons: GAM *vs.* EUR; MKK *vs.* EUR; and MKK *vs.* EUR. Here, we describe our method for assessing evidence of negative selection in the GAM *vs.* EUR cohorts. The corresponding tests for the other 2 cross-cohort comparisons proceed in a similar manner.

For each SNP and in each cohort, we recorded the SNP MAF, where the minor allele is defined as the less frequent allele in the EUR population. Note that by applying this parameter, the functional variant known to increase dependence on dietary choline is the minor allele for all 3 CD SNPs in all cohorts. We next calculated the change in MAF for SNP *j* as
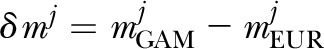
where *m*_GAM_ and *m*_EUR_ are the minor allele frequencies in the GAM and EUR populations, respectively. The mean change in MAF for a set *S* of 3 SNPs is then given by
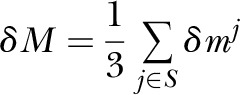
The distribution of this test statistic under the null, where all SNPs are subject to the same random fluctuations, is obtained by calculating the mean change in MAF for all 862,924 possible combinations of 3 SNPs drawn from the complete set of 174 markers. A significance measure for the alternative hypothesis that the 3 CD SNPs are under negative selection may then be computed as the proportion of all possible values for the test statistic that show a mean decrease in MAF at least as small as δ*M*^CD^, where δ*M*^CD^ is the value of δ*M*, when *S* is the set of CD SNPs. An implicit assumption is that all SNPs are independent, and, for this reason, in a preprocessing step, we filtered SNPs by LD, ensuring maximum pairwise LD *r*^2^ = 0.8. The accuracy of our method is particularly sensitive to violations of nonindependence at CD SNPs, and we therefore present the pairwise *r*^2^ coefficients for these in **Table 1**.

**TABLE 1.** Pairwise *r*^2^ coefficients for 3 CD SNPs in each cohort

SNPs	*r*^2^(GAM)	*r*^2^ (EUR)	*r*^2^ (MKK)
rs12325817*^a^*, rs2236225	0.020	0.024	0.003
rs2236225, rs12676*^a^*	0.008	0.006	0.028
rs12325817*^a^*, rs12676*^a^*	0.003	0.000	0.005

a MKK imputed allele.

We calculated a variance-adjusted probability to account for differences in the distribution of minor allele dosage at each SNP, by computing a Welch-type *t* statistic for the mean change in MAF at SNP *j* as
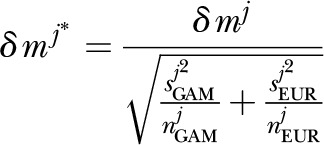
where *s^j^*_GAM_ is the sample variance in minor allele dosage in the Gambian cohort, *n^j^*_GAM_ is the number of recorded genotypes for SNP *j*, and so on. This calculation allows us, for example, to down-weight large changes in MAF between cohorts where variance in minor allele dosage within one or both cohorts is large, or the number of genotyped SNPs is relatively small. Variance-adjusted significances are then calculated by permutation as outlined above, with
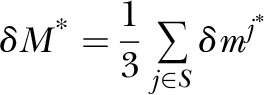
Summary statistics for all SNPs are presented in Supplemental Table 2.

#### Method 2: population genetic model

This test calculates the probability of observing the sampled data based on a standard population genetics model that assumes no selection (44). The setup and model are very similar to that described in Beaumont and Balding (45), differing only in the mechanistic details of inference. In particular, the model assumes that the 3 populations originate from a common ancestral population, equivalent to a tree merging the 3 groups *via* 2 internal nodes, with SNP allele frequencies changing from generation to generation, as they are subject to processes of random drift. In addition to allowing a joint comparison of the allele frequencies across all 3 cohorts at once, this test is expected to be more powerful than the method 1 test if the underlying model is an accurate summary of the real historical processes affecting the populations’ allele frequencies.

As in method 1, we define the minor allele to be the less frequent allele in the EUR population. At each SNP, we assume the minor allele count *X* in a given cohort (*i.e.,* where *X* 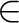 {G, C, M}, where G = GAM, C = EUR, M = MKK) follows a binomial (*n_X_*,*p_X_*) distribution, with *n_X_* the number of nonmissing sampled haplotypes and *p_X_* the (unknown) frequency of the minor allele for the given population at this SNP. As in Balding and Nichols (44), we assumed that *p_X_* follows a β distribution with mean (*p_X_*) = *p*_A_ and Var(*p_X_*) = *d_X_* *p*_A_ (1 − *p*_A_). Here, *p*_A_ is the (unknown) ancestral allele frequency for this minor allele, equivalent in this 3-population case to the allele frequency at the junction in the tree where all 3 populations merge, and *d_X_* measures the relative drift in population X from this ancestral frequency. In this scenario, we can integrate out *p_X_* analytically, giving Pr(X | *p*_A_, *d_X_*) which follows a β-binomial distribution. At the given SNP, the joint probability of the minor allele counts for all 3 cohorts, conditional on the *d_X_* of each, is:
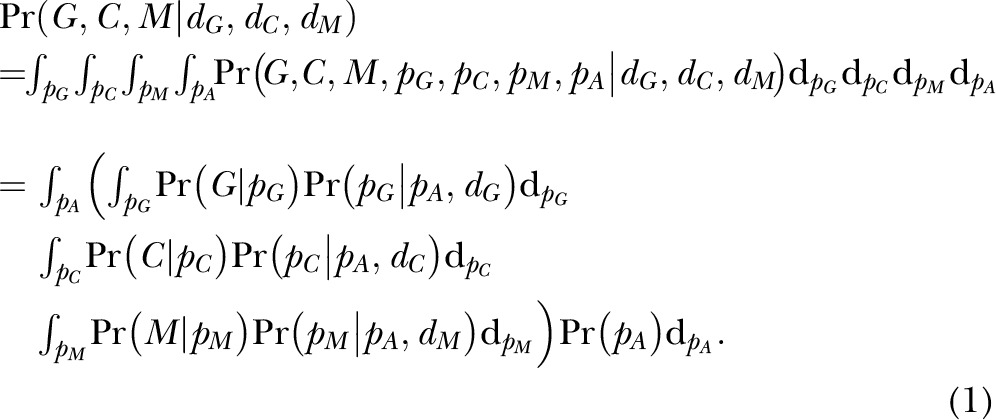
We assume Pr(*p*_A_) follows a uniform distribution and integrate out *p*_A_ numerically to calculate Eq. 1. Assuming independence across the 174 SNPs remaining after our LD-pruning procedure (see SNP filtering, above), we find the maximum likelihood estimates (MLEs) of {d_G_, d_C_, d_M_} by maximizing the joint likelihood of Eq. 1 across all 174 SNPs over a 3-dimensional grid. Assuming that the frequencies at most of these 174 SNPs are not affected by selection, this method provides estimates of the genome-wide expected drift value for each population's allele frequency relative to the ancestral frequency value, under a neutral model with no selection. Letting 
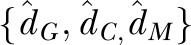
 be our maximum likelihood values of 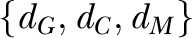 we next use Eq. 1 to calculate:
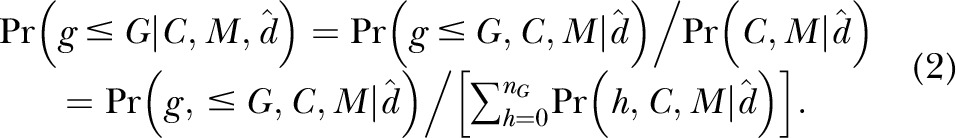
for each of 144 LD-pruned SNPs with nonmissing data in all 3 cohorts. For each SNP, this calculation gives the probability of observing a minor allele count less than or equal to that sampled in the GAM cohort, given the minor allele counts sampled in the EUR and MKK cohorts and our inferred drift values for each population. We next take the average of Eq. 2 across the 3 CD SNPs. Finally, analogous to the permutation procedure in method 1, we found the average across all 
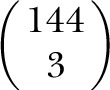
 = 487,344 subsets of 3-SNP combinations and calculated the proportion of such 3-SNP averages that were smaller than those of the 3 CD SNPs. This proportion provided an empirical probability that tested the null hypothesis that the allele frequencies for GAM at the 3 CD SNPs follow the above neutral beta-binomial model *vs.* the 1-sided alternative model in which the CD SNP GAM frequencies are smaller than that expected under the neutral model. Full details are given in Supplemental Methods 1.

## RESULTS

**Figure 2.**
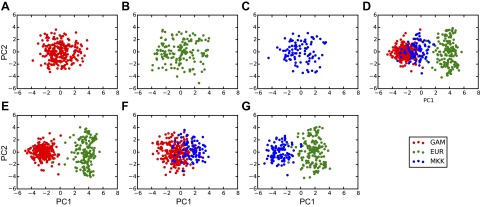
Cross-cohort comparisons confirm that GAM and MKK individuals are more closely related genetically than are EUR individuals. Plots illustrate the first 2 principal components from (*A*–*C*) 1-, (*E*–*G*) 2-, or (*D*) 3-cohort PCAs. PCAs illustrate interindividual differences at 144 SNPs across the 3 cohorts.

Principal component analyses (PCAs) of 144 SNPs in common across the 3 cohorts (GAM, EUR, and MKK) revealed the extent to which these vary in their genetic background (**Fig. 2**). The results support our hypothesis that EUR and MKK represent interesting choline-rich comparator populations, one (MKK) with a genetic background similar to that of GAM and the other (EUR) with a genetic background that is more distinct.

**Figure 3.**
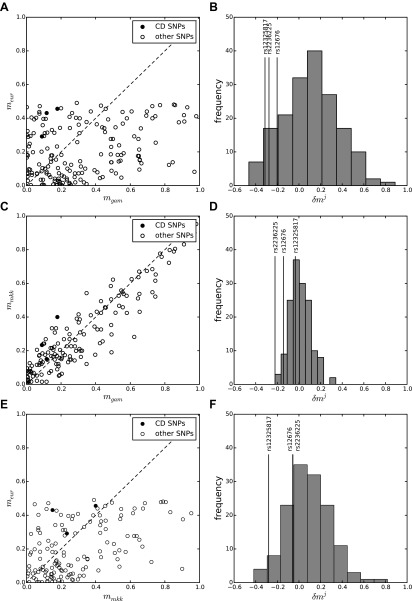
Cross-cohort MAF distributions illustrate MAF differences at CD SNPs compared to genetic background. MAF comparisons are shown for (*A*) GAM *vs.* EUR, (*C*) GAM *vs.* MKK, and (*E*) MKK *vs.* EUR. Note that the minor allele is defined for the EUR cohort, so that, in the top and bottom plots, the EUR MAF, *m*_EUR_ ≤ 0.5 for all SNPs, and the possible change in MAF for SNP *j* in the non-EUR cohort ranges from −0.5 to 1. SNPs with reduced MAF in (*A*, *C*) GAM and (*E*) MKK are located to the left of the dashed black line of parity. These include the 3 CD SNPs (filled circles). *B*, *D*, *F*) distribution of MAF differences, δ*m^j^* for each cross-cohort comparison. In each case, δ*m^j^* is defined as the SNP MAF in the cohort on the *y*-axis subtracted from the SNP MAF for the cohort on the *x*-axis. Solid black vertical lines illustrate δ*m^j^* for the 3 CD SNPs.

We first assessed evidence of negative selection of CD SNPs by using a method based on pairwise cross-cohort comparisons (method 1). We compared MAFs in GAM *vs.* EUR, GAM *vs.* MKK, and MKK *vs.* EUR. Cross-cohort MAF distributions are presented in **Fig. 3**. This figure shows a wide distribution in MAF differences across all tested SNPs in each cross-cohort comparison, although these differences are markedly reduced when the more genetically similar African populations are compared (middle plots). The 3 CD SNPs (black filled circles) show a lower MAF (negative δ*m^j^*) in GAM compared to EUR (top right plot). MAFs for CD SNPs in each cohort are presented in **Table 2**. Results of statistical tests for evidence of negative selection at CD SNPs are presented in **Table 3**. These provide strong evidence of negative selection of CD SNPs in GAM compared with both EUR and MKK (adjusted *P* = 0.007, 0.002), and weaker evidence of negative selection in MKK compared with EUR (adjusted *P* = 0.04). The evidence of negative selection was strongest in GAM *vs.* MKK, because the observed reductions in CD SNP MAFs took place against a genetic background where there was relatively little overall difference in MAFs between the 2 cohorts (Fig. 2 and middle right-hand plot in Fig. 3). Right-hand plots in Fig. 3 reveal that the 3 CD SNPs showed a relatively large reduction in MAF compared to background in GAM *vs.* EUR, whereas only rs2236225 (*MTHFD1*) and rs12676 (*CHDH*) showed such a reduction in GAM *vs.* MKK and only rs12325817 (*PEMT*) in MKK *vs.* EUR.

**TABLE 2.** Minor allele frequencies at 3 CD SNPs in the GAM, MKK, and EUR cohorts

SNPs	Minor (major) allele from EUR data	MAF_GAM	MAF_MKK	MAF_EUR
rs12676*^a^*^,^*^b^*	T(G)	0.09	0.23	0.29
rs2236225	A(G)	0.18	0.40	0.46
rs12325817*^a^*^,^*^b^*	C(G)	0.12	0.15	0.43

a MKK-imputed allele.

b Reported based on the reverse genome strand, because these genes are transcribed from that strand (dbSNP build 141).

**TABLE 3.** Statistical tests for evidence of negative selection at 3 CD SNPs, according to cross-cohort comparison method 1

Comparison	Null hypothesis tested	SNPs tested (*n*)	Unadjusted *P*	Variance-adjusted *P*
GAM *vs.* EUR	CD SNP MAFs are not significantly reduced in GAM compared with EUR	174	0.004	0.007
GAM *vs.* MKK	CD SNP MAFs are not significantly reduced in GAM compared with MKK	149	0.002	0.002
MKK *vs.* EUR	CD SNP MAFs are not significantly reduced in MKK compared with EUR	141	0.03	0.04

We performed a further, independent test to identify negative selection of CD SNPs by using an alternative population genetic model that compares SNP frequencies across all 3 cohorts simultaneously (method 2). Results are presented in **Table 4**. Although this method tests a slightly different null hypothesis—namely, whether the GAM data at the CD SNPs follow expectations under a neutral model, given the EUR and MKK data—the results strongly support the findings of method 1. In particular, we found strong evidence of negative selection of CD SNPs in GAM than in EUR and MKK (*P* = 0.008 by permutation) and no evidence of negative selection in MKK *vs.* EUR and GAM (*P* = 0.7).

**TABLE 4.** Statistical tests for evidence of negative selection at 3 CD SNPs, according to population genetic model-based method 2

Comparison	Null hypothesis tested	SNPs tested (*n*)	Permutation *P*
GAM *vs.* (EUR+MKK)	CD SNP MAFs are not significantly reduced in GAM compared with EUR and MKK	144	0.008
MKK *vs.* (EUR+GAM)	CD SNP MAFs are not significantly reduced in MKK compared with EUR and GAM	144	0.7

## DISCUSSION

Choline deficiency has known deleterious effects on health (3, 6–11) and reproduction (20). Although the essentiality of choline in the diet has been tested directly only in U.S. populations where the 2 most prominent races are Caucasian and African American (3–6), the biologic consequences of inadequate choline intake have been demonstrated in a wide range of human (3, 6–11) and rodent studies (46–48). It is therefore biologically plausible that where dietary choline is restricted, a genetically optimized choline metabolism would be likely to confer a survival advantage, irrespective of ethnicity or geographic location.

Numerous statistical methods have been developed for identifying genomic regions undergoing selection (see Ref. 49 for a review). Several rely on capturing multiple variants within each locus (50–52) or require densely genotyped data (53, 54). In this study, we instead used 2 independent statistical methods that enable analysis of sparsely genotyped unlinked SNPs, to assess for evidence of negative selection of SNPs that increase dependence on dietary choline in populations with divergent access to choline-containing foods. The first method used a standard statistical test based on cross-cohort comparisons of observed differences in allele frequencies in the GAM, EUR, and MKK cohorts, and compared MAF changes in known CD SNPs against other genotyped SNPs not known to affect dependence on dietary choline. In the second method, we modeled observed MAF differences in a population genetic model closely related to work described by Beaumont and Balding (45) that describes processes of drift that lead to genetic divergence between populations over time. As in method 1, we assumed that the non-CD SNPs are neutral, both when inferring the relative levels of drift separating populations’ allele frequencies and when generating an empirical null distribution to calculate probabilities. It remains a possibility that 1 or more of these “background” SNPs could influence dependence on dietary choline, potentially biasing our test statistics in one or the other direction, depending on whether minor alleles increase or decrease this dependence. Indeed, an interesting finding that warrants further investigation is the presence of other SNPs that have very high or very low MAFs in GAM vs. EUR (Fig. 3*A*, top left and bottom right quadrants). These represent promising candidates for future functional studies. Both statistical approaches assume that SNPs are independent after LD pruning, although we note that results changed little when no SNPs were excluded based on LD.

The population in GAM is a good model for low choline availability. Our study cohort is from the Kiang West district in rural Gambia, where mean choline intake in women was recently estimated to be 155 mg/d, with only 2.8% of the women consuming intakes above 425 mg/d (24). This level of intake is in line with historic evidence and documentation describing the traditional Gambian diet, which is rice-based and low in choline-rich foods, such as meats, milk, and eggs (25–28). In contrast, in the U.S. choline-rich foods are abundant in the current food supply, and the mean choline intake is ∼2 times higher than in The Gambia (32, 55). Investigations of traditional foods in the United States suggest an abundance of foods of animal origin (30, 31), which supports the likelihood of higher choline availability in Caucasian immigrant populations in the United States than in GAM during evolutionarily relevant time frames. It is notable that current intakes of choline in Europe are similar to those in the United States (56) and are in agreement with traditional foods consumed in Europe (57). Therefore, although there is a lack of direct evidence on historic diets, current intakes in GAM, the United States, and Europe align with traditional diets and support our characterization of low choline intake in GAM relative to that in the United States and Europe. Despite the inherent difficulty in characterizing historic diets in evolutionary studies, there is evidence supporting recent and continuous diet-driven selection in humans (58). Although we focused on dietary choline because of the known effects of choline deficiency in humans and the modulation of these effects by specific genetic variants, we acknowledge the possibility that other 1-carbon nutrients could influence the negative selective pressure that we addressed in this study.

Our evidence that negative selection occurs at 3 functional CD SNPs in different genes that independently modulate choline metabolism supports our hypothesis that the observed MAF changes are unlikely to have occurred by chance. These findings were strengthened by observed shifts in MAF in MKK, a population that is genetically similar to GAM (59, 60), but with a traditionally much higher intake of choline from foods such as milk, meat, and blood (33). It is therefore striking that a cross-cohort comparison of GAM *vs.* MKK provided equally strong evidence of negative selection at CD SNPs, supporting the argument that MAF differences are due to differences in choline intake, rather than chance or some other factor. Our use of MKK HapMap genotypes required that we impute multiple missing SNPs to enable a comparison with existing EUR and GAM data. Genotype imputation is an established method for inferring missing genotypes, although imputation accuracy can vary between populations and genomic regions (61). Internal cross-validation checks confirmed that imputation of missing genotypes for MKK data was successful. We note that neither of our statistical methods is able to distinguish between the equivalent scenarios of negative selection of CD SNPs in GAM and positive selection of CD SNPs in MKK and EUR. However, given the known deleterious effects of these SNPs in conditions of low dietary choline, we consider the former scenario to be the most probable.

The results presented here are consistent with those in other studies showing the influence of diet on gene selection. A prominent example is the genotype-mediated persistence of lactase functionality, and thus the ability to digest lactose in milk, in populations with high dairy intake such as the Maasai (62). It is interesting that this persistence occurs in parallel with positive selection of lipid metabolism gene variants that are cardioprotective (63). In this population, the high cholesterol and fat intake from the traditional diet is not accompanied by the high blood cholesterol levels and increased incidence of cardiovascular disease that is seen in European populations where lactase function persists in the absence of the positive selection of lipid metabolism variants and in an environment where high fat, high cholesterol foods are common (63). This suggests that the mismatch between diet and the genes involved in the metabolic pathways of these dietary components in Europeans contribute to adverse health outcomes. The selection for lactase persistence is estimated to have occurred 7500 years ago, suggesting that relatively recent dietary influences can modify the persistence of genetic variants (64). Additional support for the influence of diet on genetic variation is the positive selection in populations with high starch intake of additional copies of the salivary amylase gene which encodes the enzyme responsible for starch hydrolysis (65). The switch to high-starch diets occurred approximately 10,000 years ago after the transition from hunter-gathering to farming, providing additional support for the influence of relatively recent dietary exposures on the genome (66). These diet-genome interactions are believed to optimize metabolic requirements in humans (67), which fits with our hypothesis that in The Gambia, choline metabolism was genetically optimized to adjust for a diet low in sources of choline.

In this study, low dietary choline correlated with a reduced frequency of alleles that increase dependence on dietary choline. This finding could have health implications if there is a mismatch between choline intake and a population’s endogenous capacity to produce choline and its metabolites. For example, a recent report on food patterns in MKK shows a shift from a traditional high-choline diet composed primarily of meat, milk, and blood [which averages approximately 58 mg of choline per 100 g food (68)] to one composed primarily of milk, maize, and beans (69) [which averages about 15 mg choline per 100 g food (68)]. This shift could have health consequences for future generations of Maasai, whose genotypes are adapted to a high-choline diet. Our finding that SNPs that influence choline requirements occur at different frequencies across populations raises the possibility that current recommended intake levels for choline are not optimal across all populations and that they may need to be reevaluated to account for genetic differences. Finally, current methods for identifying functional genetic variants are labor and cost intensive, involving computationally intensive genome-wide screens combined with large epidemiologic studies or in-depth phenotyping in clinical studies. In this study, we offer a relatively simple alternative approach, whereby differences in the frequency of genetic variants within nutrient-relevant metabolic pathways across populations with divergent levels of nutrient intake can highlight putative functional SNPs that warrant further investigation.

## Supplementary Material

Supplemental Data

## Abbreviations

AI: adequate intake
CD: choline dependence/dependent
*CHDH*: choline dehydrogenase
EUR: European
GAM: The Gambia
LD: linkage disequilibrium
MKK: Maasai in Kinyawa, Kenya
MALDI-TOF: matrix-assisted laser desorption/ionization–time-of-flight
MAF: minor allele frequency
*MTHFD1*: methylenetetrahydrofolate reductase 1
PCA: principal component analysis
*PEMT*: phosphatidylethanolamine-*N*-methyltransferase
SNP: single-nucleotide polymorphism
THF: tetrahydrofolate
