# Supplementary material for: Evidence for negative selection of gene variants that increase dependence on dietary choline in a Gambian cohort
Source: FASEB J. 2015 Apr 28;29(8):3426–35. doi: 10.1096/fj.15-271056 (PMC4511208; doi:10.1096/fj.15-271056)
Supplement: Supplemental Data [file supp_29_8_3426__index.html]

Evidence for negative selection of gene variants that increase dependence on dietary choline in a Gambian cohort — Supplemental Data 

# Evidence for negative selection of gene variants that increase dependence on dietary choline in a Gambian cohort

## Supplemental Data

**Files in this Data Supplement:**

- Supplemental Data
- Supplemental Data
