# Supplementary material for: Evidence for negative selection of gene variants that increase dependence on dietary choline in a Gambian cohort
Source: FASEB J. 2015 Apr 28;29(8):3426–35. doi: 10.1096/fj.15-271056 (PMC4511208; doi:10.1096/fj.15-271056)
Supplement: Supplemental Data [file supp_fj.15-271056_Supplemental_Data.pdf]

# Supplementary Methods

## Method 2

As an alternative to the pairwise comparison of populations' allele frequencies ("Method 1"), we also considered an approach based on a standard population genetics model (e.g. (1; 2)). One advantage of this alternative approach is that it compares allele frequencies simultaneously across all three populations, e.g. testing whether allele frequencies are lower in the Gambia cohort at the SNPs of interest relative to *both* the North Carolina and Maasai cohorts. This technique matches that described in (3) when fixing the values of "drift" defined below (i.e. $d_G, d_C, d_M$) to be constant across all SNPs within each population, though differs in mechanistic details for inferring these "drift" terms and other values.

Let $n_G$, $n_C$ and $n_M$ be the number of non-missing haplotypes at a given SNP for the samples from Gambia, North Carolina and the Maasai, respectively. (For notational clarity we do not index the SNP here.) Let $G \leq n_G$, $C \leq n_C$ and $M \leq n_M$ be the counts of allele type $x$, which is defined as the less frequent sampled allele at this SNP in the North Carolina samples. Furthermore, let $p_G$, $p_C$ and $p_M$ be the true (unobserved) proportion of allele type $x$ at this SNP in the Gambia, North Carolina and Maasai populations.

The counts $G, C, M$ conditional on $p_G, p_C, p_M$ (and the number of non-missing haplotypes $n_G, n_C, n_M$) are assumed to follow independent Binomial distributions, i.e.

$$\begin{aligned}
\Pr(G \mid p_G) &= \text{Binomial}(n_G, p_G), \\
\Pr(C \mid p_C) &= \text{Binomial}(n_C, p_C), \\
\Pr(M \mid p_M) &= \text{Binomial}(n_M, p_M).
\end{aligned}$$

Following (1), we assume that the frequencies for each of the three populations are related by a star-shaped phylogeny to an ancestral population that has frequency $p_A$. (I.e. $p_G, p_C, p_M$ are independent after conditioning on $p_A$. Note that in this three population set-up this simply means assuming a root at the junction in the tree where all three populations merge, and then measuring the relative drift from this root.) Under a null model of "no selection" at SNP $l$, we assume:

$$\begin{aligned}
\Pr(p_G \mid p_A, d_G) &= \text{Beta}(p_A \tfrac{1-d_G}{d_G}, (1-p_A)\tfrac{1-d_G}{d_G}), \\
\Pr(p_C \mid p_A, d_C) &= \text{Beta}(p_A \tfrac{1-d_C}{d_C}, (1-p_A)\tfrac{1-d_C}{d_C}), \\
\Pr(p_M \mid p_A, d_M) &= \text{Beta}(p_A \tfrac{1-d_M}{d_M}, (1-p_A)\tfrac{1-d_M}{d_M}),
\end{aligned}$$

with $d_G$, $d_C$ and $d_M$ measuring the level of relative amount by which Gambia, North Carolina and the Maasai are drifted from this hypothetical ancestral population. Note that under this formulation, $p_G$ has mean $p_A$ and variance $d_G p_A(1-p_A)$, so that $d_G \in (0,1)$ measures the factor decrease in variance when predicting $p_G$ from $p_A$, with analogous interpretations of $d_C$ and $d_M$ for their respective populations.

Finally we assume

$$\Pr(p_A) = \text{Uniform}(0,1),$$

i.e. we do not make any assumption about the ancestral population's frequency $p_A$. Then we have:

$$\begin{aligned}
\Pr(G, C, M \mid d_G, d_C, d_M) &= \int_{p_G}\int_{p_C}\int_{p_M}\int_{p_A} \Pr(G, C, M, p_G, p_C, p_M, p_A \mid d_G, d_C, d_M) \mathrm{d}p_G \mathrm{d}p_C \mathrm{d}p_M \mathrm{d}p_A \\
&= \int_{p_A} \Big( \int_{p_G} \Pr(G \mid p_G) \Pr(p_G \mid p_A, d_G) \mathrm{d}p_G \\
&\qquad \int_{p_C} \Pr(C \mid p_C) \Pr(p_C \mid p_A, d_C) \mathrm{d}p_C \\
&\qquad \int_{p_M} \Pr(M \mid p_M) \Pr(p_M \mid p_A, d_M) \mathrm{d}p_M \Big) \Pr(p_A) \mathrm{d}p_A.
\end{aligned}$$

We can integrate $\Pr(G \mid p_G)\Pr(p_G \mid p_A, d_G)$ over $p_G$, giving a beta-binomial probability for $\Pr(G \mid p_A, d_G)$, and do the analogous for $C$ and $M$, giving:

$$\begin{aligned}
\Pr(G, C, M \mid d_G, d_C, d_M) &= \int_{p_A} \Bigg( \left[ \binom{n_G}{G} \frac{\Gamma(G+p_A\frac{1-d_G}{d_G})\Gamma(n_G-G+(1-p_A)\frac{1-d_G}{d_G})}{\Gamma(n_G+\frac{1-d_G}{d_G})} \frac{\Gamma(\frac{1-d_G}{d_G})}{\Gamma(p_A\frac{1-d_G}{d_G})\Gamma((1-p_A)\frac{1-d_G}{d_G})} \right] \\
&\quad \left[ \binom{n_C}{C} \frac{\Gamma(C+p_A\frac{1-d_C}{d_C})\Gamma(n_C-C+(1-p_A)\frac{1-d_C}{d_C})}{\Gamma(n_C+\frac{1-d_C}{d_C})} \frac{\Gamma(\frac{1-d_C}{d_C})}{\Gamma(p_A\frac{1-d_C}{d_C})\Gamma((1-p_A)\frac{1-d_C}{d_C})} \right] \\
&\quad \left[ \binom{n_M}{M} \frac{\Gamma(M+p_A\frac{1-d_M}{d_M})\Gamma(n_M-M+(1-p_A)\frac{1-d_M}{d_M})}{\Gamma(n_M+\frac{1-d_M}{d_M})} \frac{\Gamma(\frac{1-d_M}{d_M})}{\Gamma(p_A\frac{1-d_M}{d_M})\Gamma((1-p_A)\frac{1-d_M}{d_M})} \right] \Bigg) \\
&\quad \Pr(p_A)\mathrm{d}p_A \\
&\approx \frac{1}{J-1}\sum_{j=1}^{J-1} \Bigg( \left[ \binom{n_G}{G} \frac{\Gamma(G+(\frac{j}{J})\frac{1-d_G}{d_G})\Gamma(n_G-G+(1-\frac{j}{J})\frac{1-d_G}{d_G})}{\Gamma(n_G+\frac{1-d_G}{d_G})} \frac{\Gamma(\frac{1-d_G}{d_G})}{\Gamma((\frac{j}{J})\frac{1-d_G}{d_G})\Gamma((1-\frac{j}{J})\frac{1-d_G}{d_G})} \right] \\
&\quad \left[ \binom{n_C}{C} \frac{\Gamma(C+(\frac{j}{J})\frac{1-d_C}{d_C})\Gamma(n_C-C+(1-\frac{j}{J})\frac{1-d_C}{d_C})}{\Gamma(n_C+\frac{1-d_C}{d_C})} \frac{\Gamma(\frac{1-d_C}{d_C})}{\Gamma((\frac{j}{J})\frac{1-d_C}{d_C})\Gamma((1-\frac{j}{J})\frac{1-d_C}{d_C})} \right] \\
&\quad \left[ \binom{n_M}{M} \frac{\Gamma(M+(\frac{j}{J})\frac{1-d_M}{d_M})\Gamma(n_M-M+(1-\frac{j}{J})\frac{1-d_M}{d_M})}{\Gamma(n_M+\frac{1-d_M}{d_M})} \frac{\Gamma(\frac{1-d_M}{d_M})}{\Gamma((\frac{j}{J})\frac{1-d_M}{d_M})\Gamma((1-\frac{j}{J})\frac{1-d_M}{d_M})} \right] \Bigg).
\end{aligned} \tag{S1}$$

As an exact integration over $p_A$ is analytically challenging, in the last step of (S1) we approximate this integration by replacing $p_A$ with $\frac{j}{J}$ for $j \in [1, ..., J-1]$ for some large number $J$ (note that (S1) is undefined at $j = 0, J$). In practice we use $J = 1000$ for results here.

Now let $G_l$, $C_l$ and $M_l$ be the data at SNP $l$, for $l \in [1, ..., L]$ with $L$ the total number of SNPs. Here we used $L = 174$ of the total 212 SNPs that remained after an LD-pruning procedure (see "SNP Filtering" in "Methods") and were polymorphic in at least one of the three populations and non-missing in at least two of the the three cohorts. (I.e. we included 28 of the 34 SNPs that were not imputed – and hence missing – in the Maasai, and two SNPs that had genotyping rates <90% – and hence were considered missing – in either the Gambia or North Carolina cohorts.) As SNPs are assumed independent after LD-pruning, we have:

$$\mathrm{L}(d_G, d_C, d_M) = \prod_{l=1}^{L} \Pr(G_l, C_l, M_l \mid d_G, d_C, d_M). \tag{S2}$$

We maximize (S2) for $d_G, d_C, d_M$, using a 15-point equally-spaced grid for each $d_i \in [0.02, ..., 0.30]$. This gave a maximum-likelihood-estimates $\hat{d}_G, \hat{d}_C, \hat{d}_M$ of $\{0.08, 0.22, 0.04\}$. (When using all 211 SNPs with data in at least two of the three cohorts, the estimates were extremely similar: $\{0.08, 0.24, 0.04\}$.)

Our alternative hypothesis is that for a given SNP the count $G$ of allele type $x$ in the Gambian population is lower than expected under the neutral model of no selection we just derived (i.e. shows evidence for negative selection). Fixing $\hat{d} \equiv \{d_G = \hat{d}_G, d_C = \hat{d}_C, d_M = \hat{d}_M\}$, for a given SNP we can use (S1) to calculate the probability of observing $G$ or fewer haplotypes of type $x$ under the null hypothesis of no selection, for any particular values of $C, M$:

$$
\begin{aligned}
\Pr(g \leq G, C, M \mid \hat{d}) \;=\; & \sum_{g=0}^{G} \Bigg[ \int_{p_A} \Bigg( \Bigg[ \binom{n_G}{G} \frac{\Gamma(G+p_A\frac{1-\hat{d}_G}{\hat{d}_G})\Gamma(n_G-G+(1-p_A)\frac{1-\hat{d}_G}{\hat{d}_G})}{\Gamma(n_G+\frac{1-\hat{d}_G}{\hat{d}_G})} \frac{\Gamma(\frac{1-\hat{d}_G}{\hat{d}_G})}{\Gamma(p_A\frac{1-\hat{d}_G}{\hat{d}_G})\Gamma((1-p_A)\frac{1-\hat{d}_G}{\hat{d}_G})} \Bigg] \\
& \Bigg[ \binom{n_C}{C} \frac{\Gamma(C+p_A\frac{1-\hat{d}_C}{\hat{d}_C})\Gamma(n_C-C+(1-p_A)\frac{1-\hat{d}_C}{\hat{d}_C})}{\Gamma(n_C+\frac{1-\hat{d}_C}{\hat{d}_C})} \frac{\Gamma(\frac{1-\hat{d}_C}{\hat{d}_C})}{\Gamma(p_A\frac{1-\hat{d}_C}{\hat{d}_C})\Gamma((1-p_A)\frac{1-\hat{d}_C}{\hat{d}_C})} \Bigg] \\
& \Bigg[ \binom{n_M}{M} \frac{\Gamma(M+p_A\frac{1-\hat{d}_M}{\hat{d}_M})\Gamma(n_M-M+(1-p_A)\frac{1-\hat{d}_M}{\hat{d}_M})}{\Gamma(n_M+\frac{1-\hat{d}_M}{\hat{d}_M})} \frac{\Gamma(\frac{1-\hat{d}_M}{\hat{d}_M})}{\Gamma(p_A\frac{1-\hat{d}_M}{\hat{d}_M})\Gamma((1-p_A)\frac{1-\hat{d}_M}{\hat{d}_M})} \Bigg] \Bigg) \\
& \Pr(p_A)\mathrm{d}p_A \Bigg] \\
\approx\; & \sum_{g=0}^{G} \Bigg[ \frac{1}{J-1} \sum_{j=1}^{J-1} \Bigg( \Bigg[ \binom{n_G}{G} \frac{\Gamma(G+(\frac{j}{J})\frac{1-\hat{d}_G}{\hat{d}_G})\Gamma(n_G-G+(1-\frac{j}{J})\frac{1-\hat{d}_G}{\hat{d}_G})}{\Gamma(n_G+\frac{1-\hat{d}_G}{\hat{d}_G})} \frac{\Gamma(\frac{1-\hat{d}_G}{\hat{d}_G})}{\Gamma((\frac{j}{J})\frac{1-\hat{d}_G}{\hat{d}_G})\Gamma((1-\frac{j}{J})\frac{1-\hat{d}_G}{\hat{d}_G})} \Bigg] \\
& \Bigg[ \binom{n_C}{C} \frac{\Gamma(C+(\frac{j}{J})\frac{1-\hat{d}_C}{\hat{d}_C})\Gamma(n_C-C+(1-\frac{j}{J})\frac{1-\hat{d}_C}{\hat{d}_C})}{\Gamma(n_C+\frac{1-\hat{d}_C}{\hat{d}_C})} \frac{\Gamma(\frac{1-\hat{d}_C}{\hat{d}_C})}{\Gamma((\frac{j}{J})\frac{1-\hat{d}_C}{\hat{d}_C})\Gamma((1-\frac{j}{J})\frac{1-\hat{d}_C}{\hat{d}_C})} \Bigg] \\
& \Bigg[ \binom{n_M}{M} \frac{\Gamma(M+(\frac{j}{J})\frac{1-\hat{d}_M}{\hat{d}_M})\Gamma(n_M-M+(1-\frac{j}{J})\frac{1-\hat{d}_M}{\hat{d}_M})}{\Gamma(n_M+\frac{1-\hat{d}_M}{\hat{d}_M})} \frac{\Gamma(\frac{1-\hat{d}_M}{\hat{d}_M})}{\Gamma((\frac{j}{J})\frac{1-\hat{d}_M}{\hat{d}_M})\Gamma((1-\frac{j}{J})\frac{1-\hat{d}_M}{\hat{d}_M})} \Bigg] \Bigg) \Bigg].
\end{aligned}
\tag{S3}
$$

From (S1) and (S3), we can then condition on our observed values of $C, M$ and calculate:

$$
\begin{aligned}
\Pr(g \leq G \mid C, M, \hat{d}) &= \Pr(g \leq G, C, M \mid \hat{d}) / \Pr(C, M \mid \hat{d}) \\
&= \Pr(g \leq G, C, M \mid \hat{d}) / \big[ \textstyle\sum_{h=0}^{n_G} \Pr(h, C, M \mid \hat{d}) \big].
\end{aligned}
\tag{S4}
$$

We calculated (S4) for each of **rs12325817**, **rs2236225** and **rs12676**, giving values of 0.270, 0.073 and 0.101, respectively (Figure S1-left). (In an analysis using the $\hat{d}$ values estimated using all 211 SNPs that were non-missing in at least two of the three cohorts, these probabilities were very similar: $\{0.275, 0.074, 0.102\}$.)

We also calculated the probability in (S4) for each of the $L = 144$ SNPs with data in all three populations (i.e. excluding the 34 SNPs that were not imputed in the Maasai, the two additional SNPs with low genotyping rates in either the Gambia or North Carolina cohorts, and the SNPs removed during the LD-pruning procedure). Assuming any 3 sampled SNPs chosen at random are not under any selective pressure, we can generate an empirical null distribution for these probabilities averaged across any 3 SNPs under a model of no selection. I.e. analogous to the test presented in "Method 1" of the main paper, we considered all $\binom{144}{3} = 487,344$ subsets of 3 SNPs taken from the total 144, and calculated the mean value of (S4) within each subset. The mean value for SNPs **rs12325817**, **rs2236225** and **rs12676** is smaller than all but 0.0086 of these 3-SNP combinations (Figure S1-right), which is significant at $\alpha = 0.05$ to reject the null model of no selection. (This empirical $p$-value was 0.0125 when considering all $\binom{210}{3} = 1,521,520$ subsets of 3 among the total 210 SNPs that were non-missing in the Gambia cohort.) This provides evidence that, relative to other sampled SNPs, the minor allele counts at these 3 SNPs taken jointly are smaller than expectations under the neutral drift model.

# References


[1] D.J. Balding and R.A. Nichols. A method for quantifying differentiation between populations at multi-allelic loci and its implications for investigating identity and paternity. *Genetica*,

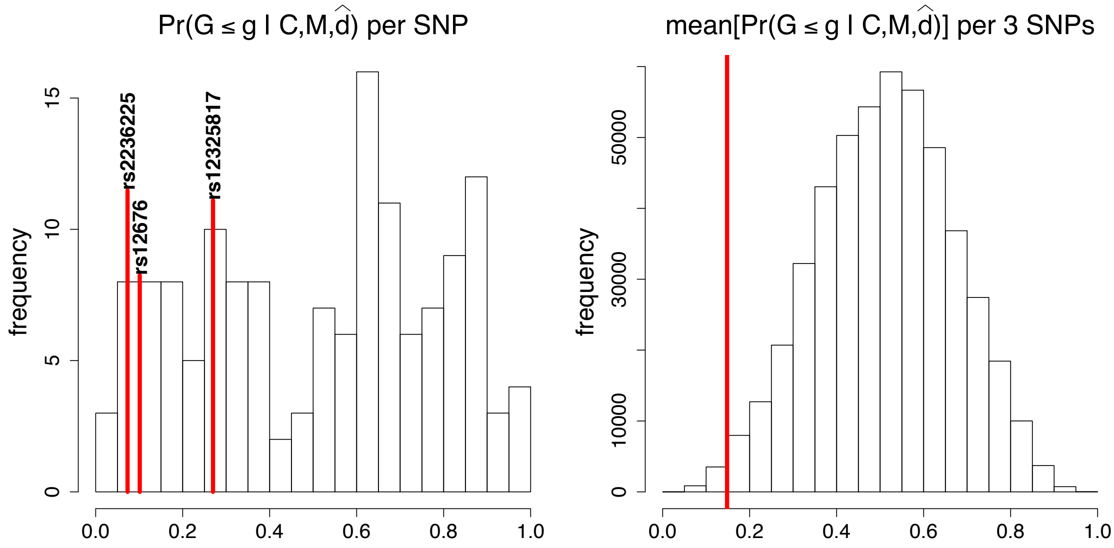


**Figure S1:** (Left) Distribution of $\Pr(g \leq G \mid C, M, \hat{d})$ from equation (S4) across all 144 non-excluded SNPs, with 3 CD SNPs highlighted in red. (Right) Distribution of $\Pr(g \leq G \mid C, M, \hat{d})$ averaged over 3 SNPs, for all $\binom{144}{3}$ 3-SNP combinations. The average for the 3 CD SNPs is highlighted in red.


96(1-2):3–12, 1995.

[2] G. Nicholson, A.V. Smith, F. Jónsson, Ó. Gústafsson, K. Stefánsson, and P. Donnelly. Assessing population differentiation and isolation from single-nucleotide polymorphism data. *Journal of the Royal Statistical Society: Series B (Statistical Methodology)*, 64:695–715, 2002.

[3] M.A. Beaumont and D.J. Balding. Identifying adaptive genetic divergence among populations from genome scans. *Molecular Ecology*, 13:969–980, 2004.
